# Genetic Variants of *lncRNA GAS5* Contribute to Susceptibility of Ischemic Stroke among Southern Chinese Population

**DOI:** 10.1155/2021/6634253

**Published:** 2021-04-10

**Authors:** Fu Deng, Peiyi Zhu, Chaoxiong Liao, Shengnan Li, Xingjuan Hu, Ying Wang, Zhaochun Wu, Shaoting Huang, Wangtao Zhong, Bin Zhao, Guoda Ma, You Li

**Affiliations:** ^1^Guangdong Key Laboratory of Age-Related Cardiac and Cerebral Diseases, Affiliated Hospital of Guangdong Medical University, Zhanjiang 524001, China; ^2^Department of Neurology, Affiliated Hospital of Guangdong Medical University, Zhanjiang 524001, China; ^3^Department of Anesthesiology, Affiliated Hospital of Guangdong Medical University, Zhanjiang 524001, China; ^4^Institute of Neurology, Affiliated Hospital of Guangdong Medical University, Zhanjiang 524001, China; ^5^Maternal and Children's Health Research Institute, Shunde Maternal and Children's Hospital, Guangdong Medical University, Shunde 528300, China

## Abstract

Emerging evidence suggests that the long noncoding RNA (lncRNA) growth arrest special 5 (GAS5) plays crucial roles in the pathogenesis of ischemic stroke (IS). The current research is aimed at assessing the correlation between two functional *GAS5* variants (rs145204276 and rs55829688) and susceptibility to IS in a Han Chinese population. This study genotyped the two *GAS5* variants in 1086 IS patients as well as 1045 age-matched healthy controls by using an improved multitemperature ligase detection reaction (iMLDR-TM) genotyping technology. We observed a considerable change in the frequencies of the rs145204276 allele and genotype among the IS patients and healthy control group. The *del*-T haplotype was substantially more prevalent in the IS cases compared to the control individuals. When study participants were stratified according to environmental factors, we found that the rs145204276 *del* allele was correlated with a higher risk of IS in male, smokers, hypertensive, and those ≥65 years old. Additional stratification conforming to IS subtypes exhibited that individuals carrying the rs145204276 *del* allele conferred a higher risk of expanding a larger artery atherosclerosis stroke subset. Moreover, there was a significant association between the rs145204276 *del* allele and elevated expression of GAS5 in IS patients. In contrast, the frequency of the allele related to rs55829688 was not statistically correlated with IS in all analysis. Our study supports a model wherein the rs145204276 variant in the *GAS5* lncRNA is associated with IS risk, thus representing a potentially viable biomarker for IS prevention and treatment.

## 1. Introduction

Stroke is known as one of the main reason of permanent disability and death all around the world [1]. Approximately 80% of strokes are ischemic in origin. Despite being a relatively common disease, the precise molecular mechanisms for the onset of ischemic stroke (IS) are still incompletely understood, with both environmental and genetic factors being implicated in this disease in a complex manner. A number of different environmental risk factors have been shown to be directly linked to IS risk, including age, gender, tobacco smoking, diabetes, hypertension, hyperlipidaemia, and hyperhomocysteinemia. In addition, a number of different genetic determinants of IS risk have been identified in genome-wide association studies, with single-nucleotide polymorphisms (SNPs) in 9p21, PITX2, HDAC9, ABO, NINJ2, ALDH2, and TSPAN2, all being linked with stroke susceptibility [2]. These SNPs, however, are still thought to account for less than half of all genetic susceptibility to IS, with a number of yet to be discovered genes also contributing to the development of IS.

Long noncoding RNAs (lncRNAs) are RNA molecules > 200 nucleotides long that do not encode proteins but regulate gene expression through a range of mechanisms, including modulation of transcriptional and posttranscriptional processing as well as modification of chromatin [3]. lncRNAs are well known to regulate a variety of biological process. Moreover, their dysregulation may lead to various neurodegenerative diseases, including Parkinson's disease [4], multiple sclerosis (MS) [5], and IS [6–8]. In particular, the lncRNA growth arrest-specific 5 (GAS5) was found upregulated in both middle cerebral artery occlusion (MCAO) rat models as well as *in vitro* cell models [9]. More recently, GAS5 inhibition was recognized as a modern therapeutic approach for ischemic brain damage due to its capability to reduce the neuronal apoptosis, abate the size of cerebral infarction, and improve neurological deficit [9, 10]. The aforementioned explanation of evidence conducted us to formulate that GAS5 may play a fundamental performance in the pathogenesis of IS.

*GAS5* is found on chromosome 1q25.1, with the gene containing 12 exons across a 4.087 kb region, coding for 29 different *GAS5* splicing variants [11]. Variants in the *GAS5* promoter region have been reported to be linked to mutiple diseases. For example, Tao et al. discovered that the rs145204276 indel polymorphism in the *GAS5* promoter area alters GAS5 transcript activity, thereby increasing hepatocellular carcinoma (HCC) risk [12]. Whereafter, Wang et al. showed that the *GAS5* rs55829688 promoter polymorphism alters the ability of the Yin-Yang 1 (YY1) transcription factor to bind to this region, thereby increasing GAS5 expression and elevated colorectal cancer risk [13]. Even more recently, Moradi et al. found that in an Iranian population the *GAS5* rs55829688 polymorphism was linked to an elevated risk of MS development [14]. However, the correlation between *GAS5* variants and the risk of IS is insufficiently investigated. The regulatory role of GAS5 in cerebral ischemic injury and its high level of expression in IS models suggest that variants in the *GAS5* gene may potentially contribute to the disease risk. In the present investigation, we therefore utilized a case-control approach to ascertain whether the two functional variants within the *GAS5* promoter region are correlated with IS susceptibility in a Southern Chinese population.

## 2. Materials and Methods

### 2.1. Participant Recruitment

The present study consecutively recruited 1086 IS patients and 1045 age-matched control participants from the Affiliated Hospital of Guangdong Medical University between 2015 and 2019. Patients with IS were independently diagnosed by clinical symptoms, computed tomography (CT) scans, and magnetic resonance imaging (MRI) analysis. The IS patients were categorized into different subsets, such as large-artery atherosclerosis (LAA) and small-artery occlusion (SAO), cardioembolic (CE), and unspecified aetiology (UE) based on the Trial of ORG 10172 in acute stroke treatment (TOAST) categorization system [15]. Patients with a history of cerebral, transient ischemia, or subarachnoid hemorrhage, autoimmune diseases, coronary artery maladies, systemic inflammatory maladies, hematological maladies, chronic infections, and malignant tumors were excluded from the study. The controls presented in this trial did not have a history of IS, chronic inflammation, autoimmunity, or the tumors with malignant status. Hypertension, diabetes, and smoking were defined based on previous criteria [16]. The investigation was confirmed *via* the Ethics Committee of the Affiliated Hospital of Guangdong Medical College. The letter of informed consent was gained from each participant prior to investigation enlistment.

### 2.2. SNP Selection and Genotyping

The two *GAS5* variants (rs145204276 and rs55829688) were selected according to prior research [12, 17]. Genomic DNA was extracted from the peripheral blood leukocyte samples using a DNA purification kit (Sangon Biotech, China). The genotyping for rs145204276 and rs55829688 was performed through an iMLDR-TM approach with the following primers, forward: 5′-ACACGACGCTCTTCCGATCTCCCTCAATCTTCCTCTCATCCAGTATCA-3′; reverse: 5′- TTCCTTGGCACCCGAGAATTCCAGGAAGGAAATCACTCAGCCTTACACC-3′. Polymerase chain reaction (PCR) was utilized to facilitate genotyping as described in previous publication [18].

### 2.3. RT-PCR

Peripheral blood mononuclear cells (PBMCs) were isolated from the whole blood by the density gradient approach of centrifugation by implementing LymphoprepTM (Axis-Shield PoCAS, Oslo, Norway) as we described previously [18]. The whole of cellular RNA from PBMCs was extracted using RNAprep pure Blood (TianGen Biotech, Beijing, China) according to the manufacturer's instructions. Total RNA was converted to cDNA utilizing the cDNA Synthesis Kit RevertAid (Thermo) conforming to the instructions of the manufacturer. Real-time PCR (RT-PCR) was then applied to quantify GAS5 expression levels with a Roche Light Cycler 480 machine and the considered primer pair: 5′- CTTCTGGGCTCAAGTGATCCT-3′ and 5′- TTGTGCCATGAGACTCCATCAG-3′. *GAPDH* served as a control for normalization purposes, using the following primer pair: 5′-GTCAACGGATTTGGTCTGTATT-3′ and 5′-AGTCTTCTGGGTGGCAGTGAT-3′. The relative expression levels of GAS5 and GAPDH were measured according to the triplicate results, and relative gene expression was calculated by the 2^-*ΔΔ*Ct^ method.

### 2.4. Statistical Analysis

The statistical tests were carried out by utilizing SPSS v19.0 (SPSS Inc., IL, USA). Chi-squared tests were used to assess the Hardy-Weinberg equilibrium (HWE) and to inquire into the classification outcomes. Consecutive information was studied *via* Student's *t*-tests. Mann–Whitney *U* test was used if the information was not distributed as normal. The frequencies of the allele and genotype related to the lncRNA *GAS5* variants between the control subjects and IS patients were measured and compared by utilizing Fisher's exact test or Chi-squared test. Haplotype assessments were performed by implementing the SHEsis computer program (http://analysis.bio-x.cn/myAnalysis.php). The relationship between specific variants and IS risk was evaluated *via* using odds ratios (ORs) and 95% confidence intervals (CIs) after modifying various criteria such as gender, age, hypertension, smoking, diabetes mellitus, and hyperlipidaemia. Bonferroni's correction was used for various comparisons with considering control type 1 error. *P* < 0.05 was the significance threshold.

## 3. Results

### 3.1. Demographic Characteristics

The demographic and clinical characteristics of the 2131 participants (1086 IS patients and 1045 healthy controls) are presented in Table 1. There was no significant difference between IS patients and controls in age (65.3 ± 9.3 vs. 65.5 ± 8.2), as well as in the levels of low-density lipoprotein (LDL), serum uric acid, and of the total amount of cholesterol. However, significant differences were observed in sex, smoking status, diabetes, and hypertension between the IS group and the controls. In the IS group, the triglyceride and homocysteine (HCY) levels tended to be higher than those observed in the controls, whereas high-density lipoprotein (HDL) cholesterol levels were lower at admission.

### 3.2. Association between the lncRNA GAS5 Variants and the Risk of IS

The allele and genotype frequencies related to the *GAS5* variants for IS patients and healthy controls are compiled in Table 2. No selection bias was evident in either groups as per the Hardy-Weinberg equilibrium test (*P* > 0.05). The distributions of the rs145204276 variant, however, varied substantially between the IS patients and control groups (*P* = 0.0020). In a dominant model (DD+ID vs. II), a considerable change was found in the frequency of the rs145204276 variant in the IS patients in comparison with that in the controls (OR = 1.36, 95% CI: 1.14–1.61, *P* = 0.0020). In addition, a substantial change was found in the recessive model frequency (II+ID vs. DD) in the IS group in comparison with that in the controls (OR = 0.70, 95% CI: 0.53–0.93, *P* = 0.026). The frequency of the variant *del* allele at rs145204276 was remarkably diverse in the IS patients compared with that in the controls (OR = 1.27, 95% CI: 1.12–1.45, *P* = 0.0016). Those participants carrying the rs145204276 del allele also exhibited increased IS risk relative to carriers of the *ins* allele (OR = 1.27, 95% CI: 1.12–1.45, *P* = 0.0016). In contrast, the distributions of the allele and genotype related to the rs55829688 variant were comparable between IS patients and control groups (*P* > 0.05). Logistic regression analysis was employed to determine the effect of some variables on IS risk. We found that the independent risk factors of IS were smoking (OR = 2.27; 95% CI, 1.53-3.38), hypertension (OR = 6.05; 95% CI, 4.49-8.13), diabetes (OR =1.82; 95% CI, 1.17-2.82), triglycerides (OR = 1.16; 95% CI, 1.06-1.27), HDL-cholesterol (OR = 0.45; 95% CI, 0.30-0.68), and rs145204276 (OR = 2.27; 95% CI, 1.30-3.95) (Table S1).

### 3.3. Haplotype Analysis

The frequency of the *del*-T haplotype (according to the rs145204276–rs55829688 variants) was significantly higher in IS patients compared with controls (OR = 1.27, 95% CI: 1.12–1.45, *P* = 6.0 × 10^−4^), and this haplotype was associated with an increased risk of IS following the adjustment of sex, age, hypertension, smoking, hyperlipidaemia, and diabetes mellitus (Table 3).

### 3.4. Associations between GAS5 Variants and Demographic Characteristics

We next examined the relationship among the studied *GAS5* variants and defined demographic characteristics in IS patient and control groups (Tables 4 and 5). Following the stratification of participants according to age, sex, smoking status, diabetes and hypertension, we found the rs145204276 del allele was associated with a higher risk of IS in both individuals ≥ 65 years old (*P* = 0.0018), male (*P* = 0.0050), smokers (*P* = 0.0018), and hypertensive patients (*P* = 0.0018) (Table 4). No significant differences were observed between rs55829688 variant distributions and IS risk when stratified *via* sex, age, diabetes, smoking status, and hypertension (*P* > 0.05) (Table 5).

### 3.5. Associations between GAS5 Variants and Stroke Subtypes

Moreover, to examine whether the impacts of *GAS5* variants were restricted to a particular subset, we stratified the IS patients into stroke subsets according to the TOAST categorization. As illustrated in Table 6, when the population was categorized with considering the TOAST categorization system, carriers with the rs145204276 *del* allele (*P* = 0.0010) had a higher risk of stroke of the LAA subset compared with controls. In contrast, no statistical correlations were found among the rs55829688 variant and the stroke subsets when compared with the healthy controls (Table 7) (*P* > 0.05).

### 3.6. Effect of rs145204276 Variant on GAS5 Expression

**Figure 1:**
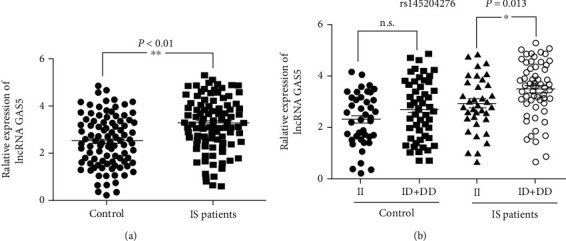
(a) Relative GAS5 expression in PBMCs from IS patients (*n* = 98) and healthy controls (*n* = 95) (^∗∗^*P* < 0.01). (b) Relative GAS5 expression in IS patients and control subjects with the rs145204276 *ins/ins* (II) and *ins/del*+*del/del* (ID+DD) genotypes. (^∗^*P* < 0.05). GAPDH served as a normalization control. The relative expression levels of GAS5 and GAPDH were measured according to the triplicate results, and relative gene expression was calculated according to the 2^-*ΔΔ*Ct^ method. IS: ischemic stroke; n.s.: not significant.

The expression levels of GAS5 were measured in the PBMCs of 98 IS patients and 95 controls (Figure 1). We found that the mean value of the GAS5 levels were significantly elevated in IS patients compared with controls (*P* < 0.01) (Figure 1(a)). In addition, when stratifying the IS patients according to rs145204276 genotypes, we observed significantly higher GAS5 expression in individuals with the rs145204276 ID+DD genotypes relative to levels in individuals with the rs145204276 II genotype (*P* = 0.013) (Figure 1(b)). No significant difference in GAS5 expression was observed in control samples when comparing the rs145204276 ID+DD and II genotypes (*P* = 0.086) (Figure 1(b)). These findings were consistent with results from the expression quantitative trait loci (eQTL). The rs145204276 DD genotype was associated with higher GAS5 expression in several tissues, such as the whole blood, frontal cortex, and artery (*P* < 0.001) (Fig.S1 A-D).

## 4. Discussion

In the current study, we present evidence that there is a significant correlation between the *GAS5* rs145204276 indel variant and IS risk in a Southern Chinese population. Haplotype analysis suggested that the *del*-T haplotype (the rs145204276–rs55829688 variants) exhibited an increased risk to IS susceptibility. Further stratification revealed that those who are ≥65 years old, male, smokers, or hypertensives, and who carry the rs145204276 *del* allele may have a higher risk of developing IS. Furthermore, IS patients bearing the rs145204276 *del* allele were linked to an increased risk of LAA stroke. In addition, we detected significantly elevated GAS5 expression in PBMCs from IS patients carrying the rs145204276 *del* allele. Aforementioned results highlight the possibility that the *GAS5* rs145204276 variant may be a valuable biomarker for predicting IS risk in certain populations.

A variety of studies showed the pivotal role of the lncRNA GAS5 in the pathophysiological processes of IS-associated neurovascular damage. Zhou et al. reported that GAS5 was upregulated in MCAO rats models and *in vitro* cell models, and it enhanced cell apoptosis in hypoxia circumstance by miR-221/PUMA axis [19]. Ying et al. showed that GAS5 knockdown improves apoptosis and inflammatory responses *via* the miR-26b-5p/Smad1 axis [20]. Deng et al. revealed that silencing GAS5 could hinder neuronal apoptosis and enhance neurological function in IS through suppressing DNMT3B-mediated MAP4K4 methylation [6]. Chen et al. reported that GAS5 could ameliorate the procedure of IS *via* accomplishing as a ceRNA for miR-137 to mediate the pathway of the Notch1 signaling [21]. In spite of these advances, the role of the *GAS5* allele in the pathophysiology of IS has not been fully explored.

Several investigations have assessed the correlation among the *GAS5* variants as well as numerous human diseases. Tao et al. previously identified the 5-bp indel rs145204276 polymorphism in the *GAS5* promoter region, which they found to be linked with elevated risk of HCC [12]. In contrast, however, Zheng et al. found this same rs145204276 polymorphism to be linked with reduced colorectal cancer risk and reduced rates of lymph node metastasis in patients with this disease [22]. In this study, we provided evidence that there was also a relationship between the *GAS5* rs145204276 indel variant and an elevated risk of IS; this outcome is in a favorable agreement with Zheng et al.'s previous report [23]. Compared to the cited study, our cohort of samples is larger, and moreover, we explored the influence of *GAS5* haplotypes, environmental factors, and stroke subtypes on IS susceptibility. Another *GAS5* promoter variant rs55829688 has been correlated with elevated risk of MS in an Iranish population [24]. However, in the current study, no significant association has been identified by comparing rs55829688 variant allele frequencies and IS risk. We further demonstrated that individuals with the *del*-T haplotype might run a higher risk of developing IS. Nevertheless, additional independent investigations are required to elucidate thoroughly the correlations between this gene and the incidence of IS within various populations.

It is widely accepted that both genetic predisposition and environmental factors contribute to IS susceptibility; however, the interaction between these variables is complex and poorly understood. There is strong epidemiological evidence indicating that age, sex, smoking, diabetes, and hypertension are all associated with IS risk [25, 26]. Aging is the strongest nonmodifiable risk factor for IS, and older people are more likely to suffer from IS, with higher mortality and poorer prognosis than their young counterparts [26]. Stroke occurs more commonly in men than in women, with the ratio of men to women for stroke incidence of about 1.3-1.5 to one [27]. Smoking and hypertension are well-known modifiable risk factors for IS. Smokers are two to three times more likely to have a stroke than nonsmokers. The outbreak of cigarette smoking is more common in men than women in China. A meta-analysis of hypertension and hazard of stroke revealed that the overall relative risk of stroke correlated with hypertension was 5.43 [28]. Consistent with these past results, when we conducted subgroup analyses of our participant cohorts, we found that IS risk was significantly increased in older, male individuals, smokers, and hypertensive individuals that are carrying the rs145204276 *del* allele. This is thus consistent with IS risk arising from a complicated interaction between genetic and environmental parameters.

Different stroke subtypes are attributable to different aetiologies, thus clarifying the IS subtypes is of importance in choosing treatment alternatives and predicting the outcome. The most likely underlying pathology of LAA stroke is atherothrombosis of large arteries [29], while the SAO stroke is attained from blockage of the penetrating arteries caused by lipohyalinosis [30], suggesting different pathogenic mechanisms of IS subtypes. In the current research, we categorized the IS patients with the TOAST classification and found that patients carrying the rs145204276 *del* allele were linked to an increased risk of stroke with LAA subtype. Our finding was partially supported by Shen and She's work, which demonstrated that the rs145204276 *del* allele significantly correlated with increased risk of atherosclerosis [31].

Functional lncRNA SNPs are those that lead to changes in either the expression or function of these lncRNAs, thereby influencing their interactions with target genes, thus potentially influencing disease risk. The *GAS5* promoter region rs145204276 indel variant has been suggested to modulate GAS5 expression as a result of alterations in *GAS5* promoter methylation [12, 32]. In addition, Tang et al. have provided evidence that the rs145204276 *del* allele is bound by the SP1 transcription factor, leading to enhanced promoter activity and consequently elevated GAS5 expression that leads to elevated breast cancer risk [33]. In a similar vein, Yuan et al. found that *GAS5* indel polymorphisms are associated with enhanced binding of TFAP2A to the promoter region, leading to increased GAS5 expression that is in turn associated with elevated glioma risk [34]. In other reports, the *GAS5* rs55829688 T>C polymorphism has been shown to alter YY1 binding to this promoter region, thereby impairing GAS5 expression and reducing the risk of colorectal cancer development [35]. Recently, Yan et al. found that the rs55829688 polymorphism enhanced the binding of transcription factor TP63, which resulted in elevated GAS5 expression, and was correlated with a poor prognosis in AML patients [36]. These evidences suggested that the rs145204276 and rs55829688 variants could regulate GAS5 expression by altering transcription factors binding with the promoter region. In this study, we found that patients with the rs145204276-mutated *del* allele exhibited higher expression of GAS5 in IS PBMCs, compared with those carrying the *ins* allele. In light of our findings and these past results, it seems likely that the rs145204276 *del* allele is associated with altered *GAS5* promoter methylation and/or transcription factor binding activity, thereby leading to altered GAS5 expression and a corresponding alteration in IS risk.

This study has several limitations. First, this was a retrospective analysis conducted in a hospital, and so there is an inherent risk of selection bias. Second, only two variants, rs145204276 and rs55829688, were assessed in this study; other variants (rs2067079, rs6790, and rs1951625) in *GAS5* may also contribute to IS risk. Third, other environmental risk factors, including the rates of smoking, diabetes, hypertension, hyperlipidaemia, and hypercholesterolemia, might have complicated the correlation between *GAS5* variants and IS. Last, the exact mechanisms whereby these *GAS5* variants influence the expression of GAS5 have yet to be firmly established. Further independent validation of our results and determination as to whether they are relevant in different ethnic backgrounds is therefore essential.

In conclusion, we demonstrated that the *GAS5* promoter region rs145204276 indel variant confers increased risk of IS in a Han Chinese population. Furthermore, the rs145204276 *del* allele may affect the genetic predisposition to this disease by increasing the expression of GAS5. However, further investigations will be necessary to validate these results and to shed light on how the *GAS5* variants influences IS onset and progression.

## Figures and Tables

**Table 1:** Characteristics of IS cases and controls.

Variables	IS patients (*n* = 1086)	Controls (*n* = 1045)	*P* value
Mean age (years)	65.3 ± 9.3	65.5 ± 8.2	0.85
Male/female	738/348	511/534	**<0.001**
Smokers, *n* (%)	304 (28.0)	125 (12.0)	**<0.001**
Hypertension, *n* (%)	828 (76.2)	341 (32.6)	**<0.001**
Diabetes, *n* (%)	366(33.7)	115(11.0)	**<0.001**
Uric acid (mmol/L)	316.5 ± 91.7	313.6 ± 89.8	0.42
Total cholesterol (mmol/L)	5.09 ± 1.03	5.11 ± 1.05	0.35
Triglycerides (mmol/L)	1.53 ± 1.06	1.42 ± 0.95	**<0.01**
HDL-cholesterol (mmol/L)	1.36 ± 0.41	1.46 ± 0.53	**<0.01**
LDL-cholesterol (mmol/L)	3.08 ± 1.03	3.03 ± 0.97	0.63
HCY (mmol/L)	10.82 ± 6.12	9.82 ± 3.45	**<0.01**

HCY: homocysteine; HDL: high-density lipoprotein; IS: ischemic stroke; LDL: low-density lipoprotein. Continuous data are presented as the mean ± standard deviation, median (range), or *n* (%). *P* < 0.05 is indicated in bold font.

**Table 2:** Frequencies of lncRNA *GAS5* genotypes and alleles in IS patients and controls.

Genotypes	IS patients, *n* = 1086 (%)	Controls, *n* = 1045 (%)	AOR (95% CI)	*P* value	*P* value^a^
rs145204276					
*ins/ins*(II)	424 (39.0)	486 (46.5)		7.3 × 10^−4^	**0.0020**
*ins/del*(ID)	528 (48.6)	465 (44.5)			
*del/del*(DD)	134 (12.3)	94 (9.0)			
Dominant model DD+ID vs. II	662 (61.0)	559 (53.5)	1.36 (1.14-1.61)	5.0 × 10^−4^	**0.0020**
Recessive model II+ID vs. DD	952 (87.7)	951 (91.0)	0.70 (0.53-0.93)	**0.013**	**0.026**
*ins* allele	1376 (63.4)	1437 (68.8)			
*del* allele	796 (36.6)	653 (31.2)	1.27 (1.12-1.45)	2.0 × 10^−4^	**0.0016**
rs55829688					
TT	579 (53.3)	517 (49.5)		0.20	0.23
TC	415 (38.2)	430 (41.1)			
CC	92 (8.5)	98 (9.4)			
Dominant model TC+CC vs. TT	507 (46.7)	528 (50.5)	0.86 (0.72-1.02)	0.076	0.12
Recessive model TT+TC vs. CC	994(91.5)	947(90.6)	1.12(0.83-1.51)	0.46	0.46
T allele	1573(72.9)	1464(70.0)			
C allele	599(27.1)	626(30.0)	0.89(0.78-1.02)	0.087	0.12

*P* value of difference in genotypes between the case group and control group. *P* < 0.05 is indicated in bold font. Adjusted for age, gender, smoking, hypertension, diabetes mellitus, and hyperlipidaemia. ^a^False discovery rate-adjusted *P* value for multiple hypotheses testing using the Benjamini-Hochberg method. AOR: adjusted odds ratio; CI: confidence interval; DD: *del/del*; ID: *ins/del*; II: *ins/ins*; IS: ischemic stroke; lncRNA: long noncoding RNA.

**Table 3:** Haplotype frequencies in cases and controls and their relationship to IS risk.

Haplotypes	Case (freq)	Control (freq)	*P* value	*P* value^a^	OR (95% CI)
*lncRNA GAS5 (rs145204276, rs55829688)*					
*del*-T	796 (36.6)	653 (31.2)	2.0 × 10^−4^	6.0 × 10^−4^	1.27 (1.12-1.45)
*ins*-C	599 (27.6)	626 (29.9)	0.087	0.087	0.89 (0.78-1.02)
*ins*-T	777 (35.8)	811 (38.8)	0.041	0.062	0.88 (0.78-1.00)

Adjusted for age, gender, smoking, hypertension, diabetes mellitus, and hyperlipidaemia. All those frequency < 0.05 will be ignored in the analysis. ^a^False discovery rate-adjusted *P* value for multiple hypotheses testing using the Benjamini-Hochberg method. *P* < 0.05 is indicated in bold font. CI: confidence interval; *del*: rs145204267 AGGCA deletion allele; freq: frequency; GAS5: growth arrest special 5; *ins*: rs145204267 AGGCA insertion allele; IS: ischemic stroke; lncRNA: long noncoding RNA; OR: odds ratio.

**Table 4:** Stratified analysis between the genotypes and alleles of *lncRNA GAS5* rs145204276 polymorphism among IS patients and the control group.

Characteristics	IS patient group					Control group					*P*_G_^a^ value	*P*_A_^a^ value
	Genotype, *n* (%)			Allele, *n* (%)		Genotype, *n* (%)			Allele, *n* (%)			
	II	ID	DD	I	D	II	ID	DD	I	D		
Age												
≥65	237 (38.5)	286 (46.5)	92 (15.0)	760 (61.8)	470 (38.2)	265 (47.1)	243 (43.2)	55 (9.8)	773 (68.7)	353 (31.3)	**0.0077**	**0.0018**
<65	187 (39.7)	242 (51.4)	42 (8.9)	616 (65.4)	326 (34.6)	221 (45.9)	222 (46.0)	39 (8.1)	664 (68.9)	300 (31.1)	0.20	0.12
Gender												
Male	281 (38.1)	359 (48.6)	98 (13.3)	921 (62.4)	555 (37.6)	232 (45.4)	234 (45.8)	45 (8.8)	698 (68.3)	324 (31.7)	**0.019**	**0.0050**
Female	142 (40.8)	170 (48.9)	36 (10.3)	454 (65.2)	242 (34.8)	254 (47.5)	231 (43.3)	49 (9.2)	739 (69.2)	329 (30.8)	0.20	0.11
Smoking												
Yes	101 (38.5)	149 (46.7)	54 (14.8)	351 (57.7)	257 (42.3)	63 (50.4)	52 (41.6)	10 (8.0)	178 (71.2)	72 (28.8)	**0.0065**	**0.0018**
No	323 (39.2)	379 (49.4)	80 (11.4)	1025 (65.5)	539 (34.4)	423 (46.0)	413 (44.9)	84 (9.1)	1259 (68.4)	581 (31.6)	0.20	0.11
Diabetes												
Yes	130 (39.6)	179 (46.4)	57 (14.0)	460 (62.8)	272 (37.2)	55 (47.8)	49 (42.6)	11 (9.6)	159 (69.1)	71 (30.9)	0.090	0.11
No	294 (38.8)	349 (49.7)	77 (11.5)	916 (63.6)	524 (36.4)	431(46.4)	416 (44.7)	83 (8.9)	1278 (68.7)	582 (31.3)	0.12	**0.0050**
Hypertension												
Yes	295 (35.6)	418 (50.5)	115 (13.9)	1008 (60.9)	648 (39.1)	158 (46.3)	152 (44.6)	31 (9.1)	468 (68.6)	214 (31.4)	**0.0065**	**0.0018**
No	129 (50.0)	110 (42.6)	19 (7.4)	368 (71.3)	148 (28.7)	328 (46.6)	313 (44.5)	63 (8.9)	969 (68.8)	439 (31.2)	0.58	0.31

DD: del/del; GAS5: growth arrest special 5; ID: ins/del; II: ins/ins; IS: ischemic stroke; lncRNA: long noncoding RNA. *P*_G_: *P* value of the difference in genotypes between the case and control groups; *P*_A_: *P* value of the difference in alleles between the case and control groups; *^a^*false discovery rate-adjusted *P* value for multiple hypotheses testing using the Benjamini-Hochberg method. *P* < 0.05 is indicated in bold font.

**Table 5:** Stratified analysis between the genotypes and alleles of *lncRNA GAS5* rs55829688 polymorphism among IS patients and the control group.

Characteristics	IS patient group					Control group					*P*_G_^a^ value	*P*_A_^a^ value
	Genotype, *n* (%)			Allele, *n* (%)		Genotype, *n* (%)			Allele, *n* (%)			
	TT	TC	CC	T	C	TT	TC	CC	T	C		
Age												
≥65	315 (50.4)	244 (40.5)	56 (9.1)	874 (71.1)	356 (28.9)	278 (49.4)	230 (40.8)	55 (9.8)	786 (69.8)	340 (30.2)	0.89	0.62
<65	264 (56.1)	171 (36.3)	36 (7.6)	699 (74.2)	243 (25.8)	239 (49.6)	200 (41.5)	43 (8.9)	678 (70.3)	286 (29.7)	0.64	0.35
Gender												
Male	390 (52.8)	289 (39.2)	59 (8.0)	1069 (72.4)	407 (27.6)	259 (50.7)	211 (41.3)	41 (8.0)	729 (71.3)	293 (28.7)	0.93	0.62
Female	189 (54.3)	126 (36.2)	33 (9.5)	504 (72.4)	192 (27.6)	258 (48.3)	219 (41.0)	57 (10.7)	735 (68.8)	333 (31.2)	0.64	0.35
Smoking												
Yes	167 (54.9)	105 (34.5)	32 (10.5)	439 (72.2)	169 (27.8)	60 (48.0)	53 (42.4)	12 (9.6)	173 (69.2)	77 (30.8)	0.64	0.62
No	412 (52.7)	310 (39.6)	60 (7.7)	1134 (72.5)	430 (27.5)	457 (49.7)	377 (41.0)	86 (9.3)	1291 (70.2)	549 (29.8)	0.64	0.35
Diabetes												
Yes	191 (52.2)	140 (38.3)	35 (9.5)	522 (71.3)	210 (28.7)	55 (47.8)	48 (41.7)	12 (10.5)	158 (68.7)	72 (31.3)	0.93	0.62
No	388 (53.9)	275 (38.2)	57 (7.9)	1051 (73.0)	389 (27.0)	462 (49.7)	382 (41.1)	86 (9.2)	1306 (70.2)	554 (29.8)	0.64	0.35
Hypertension												
Yes	442 (53.4)	316 (38.1)	70 (8.5)	1200 (72.5)	456 (27.5)	177 (51.9)	134 (42.2)	30 (8.8)	488 (71.6)	194 (28.4)	0.89	0.68
No	137 (53.1)	99 (38.4)	22 (8.5)	373 (72.3)	143 (27.7)	340 (50.0)	296 (40.6)	68 (9.4)	976 (69.3)	432 (30.7)	0.43	0.44

GAS5: growth arrest special 5; IS: ischemic stroke; lncRNA: long noncoding RNA. *P*_G_: *P* value of the difference in genotypes between the case and control groups; *P*_A_: *P* value of the difference in alleles between the case and control groups; *^a^*false discovery rate-adjusted *P* value for multiple hypotheses testing using the Benjamini-Hochberg method.

**Table 6:** The relationship between *lncRNA GAS5* rs145204276 variant and IS subtypes in IS patients.

	*lncRNA GAS5 rs145204276*							
	Genotype			*P* value^a^	Allele		*P* value^a^	OR (95% CI)
	II	ID	ID		I	D		
Control	486 (46.5)	465 (44.5)	94 (9.0)		1437 (68.8)	653 (31.2)		
*Cases*								
LAA	265 (38.5)	335 (48.6)	89 (12.9)	**0.0040**	865 (62.8)	513 (37.2)	**0.0010**	1.31 (1.13-1.51)
SAO	122 (40.5)	149 (49.5)	30 (10.0)	0.18	393 (65.3)	209 (34.7)	0.15	1.17(0.97-1.42)
CE	18 (46.2)	14 (35.9)	7 (17.9)	0.18	50 (64.1)	28 (35.9)	0.39	1.23(0.77-1.98)
UE	19 (33.3)	30 (52.6)	8 (14.1)	0.18	68 (59.6)	46 (40.4)	0.098	1.49(1.01-2.19)

CE: cardioembolic; CI: confidence interval; DD: *del/del*; GAS5: growth arrest special 5; ID: *ins/del*; II: *ins/ins*; IS, ischemic stroke; LAA: large-artery atherosclerosis; lncRNA: long noncoding RNA; OR, odds ratio; SAO: small-artery occlusion; UE: unspecified aetiology. ^a^False discovery rate-adjusted P value for multiple hypotheses testing using the Benjamini-Hochberg method. P <0.05 is indicated in bold font.

**Table 7:** The relationship between *lncRNA GAS5* rs55829688 variant and IS stratified by TOAST classification in IS patients.

	*lncRNA GAS5* rs55829688							
	Genotype			*P* value^a^	Allele		*P* value^a^	OR (95% CI)
	TT	TC	CC		T	C		
Control	517 (49.5)	430 (41.1)	98 (9.4)		1464 (70.0)	626 (30.0)		
*Cases*								
LAA	384 (56.0)	250 (36.0)	55 (8.0)	0.16	1018 (73.9)	360 (26.1)	0.064	0.83 (0.71-0.96)
SAO	154 (51.2)	119 (39.5)	28 (9.3)	0.91	427 (70.9)	175 (29.1)	0.91	0.96 (0.79-1.17)
CE	20 (51.3)	15 (38.5)	4 (10.3)	0.91	55 (70.5)	23 (29.5)	0.92	0.98 (0.60-1.61)
UE	21 (36.8)	31 (54.4)	5 (8.8)	0.26	73 (64.0)	41 (36.0)	0.34	1.31 (0.89-1.95)

CE: cardioembolic; CI: confidence interval; GAS5: growth arrest special 5; IS: ischemic stroke; LAA: large-artery atherosclerosis; lncRNA: long noncoding RNA; OR, odds ratio; SAO: small-artery occlusion; UE: unspecified aetiology. ^a^False discovery rate-adjusted *P* value for multiple hypotheses testing using the Benjamini-Hochberg method.

## Data Availability

The data are available upon reasonable request.

## Supplementary Materials

Supplementary Materials Figure S1: the correlation between rs145204276 genotype and expression of GAS5. (A) Quantitative real-time PCR was used to examine the expression levels of GAS5 in 98 IS patients. Compared to rs145204276 *ins/ins* (II) carriers, rs145204276 *del/del* (DD) carriers had increased levels of GAS5 (^∗∗^*P* < 0.01). Expression quantitative trait loci analysis of rs145204276 with gene expression in different tissues (B), frontal cortex (C), and artery (D). eQTL: expression quantitative trait loci; NES: normalized effect size; CI: confidence interval. Table S1: logistic regression analysis for identifying risk factors of IS.

## References

[1] Dmytriw A. A., Zhang Y., Mendes Pereira V. (2019). Mechanical thrombectomy and the future of acute stroke treatment. *European Journal of Radiology*.

[2] Lindgren A. (2014). Stroke genetics: a review and update. *J Stroke*.

[3] Morris K. V., Mattick J. S. (2014). The rise of regulatory RNA. *Nature Reviews. Genetics*.

[4] Xu W., Zhang L., Geng Y., Liu Y., Zhang N. (2020). Long noncoding RNA GAS5 promotes microglial inflammatory response in Parkinson's disease by regulating NLRP3 pathway through sponging miR-223-3p. *International Immunopharmacology*.

[5] Sun D., Yu Z., Fang X. (2017). LncRNA GAS5 inhibits microglial M2 polarization and exacerbates demyelination. *EMBO Reports*.

[6] Deng Y., Chen D., Gao F. (2020). Silencing of long non-coding RNA GAS5 suppresses neuron cell apoptosis and nerve injury in ischemic stroke through inhibiting DNMT3B-dependent MAP4K4 methylation. *Translational Stroke Research*.

[7] Bao M.-H., Szeto V., Yang B. B., Zhu S.-Z., Sun H.-S., Feng Z.-P. (2018). Long non-coding RNAs in ischemic stroke. *Cell Death & Disease*.

[8] Zhu R., Liu X., He Z. (2018). Long non-coding RNA H19 and MALAT1 gene variants in patients with ischemic stroke in a northern Chinese Han population. *Molecular Brain*.

[9] Zhao R. B., Zhu L. H., Shu J. P., Qiao L. X., Xia Z. K. (2018). GAS5 silencing protects against hypoxia/ischemia-induced neonatal brain injury. *Biochemical and Biophysical Research Communications*.

[10] Li J., Lv H., Che Y. Q. (2020). Long non-coding RNA Gas5 potentiates the effects of microRNA-21 downregulation in response to ischaemic brain injury. *Neuroscience*.

[11] Toraih E. A., Alghamdi S. A., El-Wazir A. (2018). Dual biomarkers long non-coding RNA GAS5 and microRNA-34a co-expression signature in common solid tumors. *PLoS One*.

[12] Tao R., Hu S., Wang S. (2015). Association between indel polymorphism in the promoter region of lncRNA GAS5 and the risk of hepatocellular carcinoma. *Carcinogenesis*.

[13] Wang Y., Wu S., Yang X., Li X., Chen R. (2019). Association between polymorphism in the promoter region of lncRNA GAS5 and the risk of colorectal cancer. *Bioscience Reports*.

[14] Moradi M., Gharesouran J., Ghafouri-Fard S. (2020). Role of NR3C1 and GAS5 genes polymorphisms in multiple sclerosis. *International Journal of Neuroscience*.

[15] Adams H. P., Bendixen B. H., Kappelle L. J. (1993). Classification of subtype of acute ischemic stroke. Definitions for use in a multicenter clinical trial. TOAST. Trial of Org 10172 in Acute Stroke Treatment. *Stroke*.

[16] Li Y., Cui L. L., Li Q. Q. (2014). Association between ADAM17 promoter polymorphisms and ischemic stroke in a Chinese population. *Journal of Atherosclerosis and Thrombosis*.

[17] Zhu L., Zhu Q., Wen H., Huang X., Zheng G. (2019). Mutations in GAS5 affect the transformation from benign prostate proliferation to aggressive prostate cancer by affecting the transcription efficiency of GAS5. *Journal of Cellular Physiology*.

[18] Li Y., Liao F., Yin X. J. (2013). An association study on ADAM10 promoter polymorphisms and atherosclerotic cerebral infarction in a Chinese population. *CNS Neuroscience & Therapeutics*.

[19] Zhou X.-B., Lai L.-F., Xie G.-B., Ding C., Xu X., Wang Y. (2020). LncRNAGAS5 sponges miRNA-221 to promote neurons apoptosis by up-regulated PUMA under hypoxia condition. *Neurological Research*.

[20] Shangguan Y., Han J., Su H. (2020). GAS5 knockdown ameliorates apoptosis and inflammatory response by modulating miR-26b-5p/Smad1 axis in cerebral ischaemia/reperfusion injury. *Behavioural Brain Research*.

[21] Chen F., Zhang L., Wang E., Zhang C., Li X. (2018). LncRNA GAS5 regulates ischemic stroke as a competing endogenous RNA for miR-137 to regulate the Notch1 signaling pathway. *Biochemical and Biophysical Research Communications*.

[22] Zheng Y., Song D., Xiao K. (2016). LncRNA GAS5 contributes to lymphatic metastasis in colorectal cancer. *Oncotarget*.

[23] Zheng Z., Liu S., Wang C., Han X. (2018). A functional polymorphism rs145204276 in the promoter of long noncoding RNA GAS5 is associated with an increased risk of ischemic stroke. *Journal of Stroke and Cerebrovascular Diseases*.

[24] Moradi M., Gharesouran J., Ghafouri-Fard S. (2020). Role of NR3C1 and GAS5 genes polymorphisms in multiple sclerosis. *The International Journal of Neuroscience*.

[25] Wu S., Wu B., Liu M. (2019). Stroke in China: advances and challenges in epidemiology, prevention, and management. *Lancet Neurology*.

[26] Roy-O'Reilly M., McCullough L. D. (2018). Age and sex are critical factors in ischemic stroke pathology. *Endocrinology*.

[27] Liu M., Wu B., Wang W. Z., Lee L. M., Zhang S. H., Kong L. Z. (2007). Stroke in China: epidemiology, prevention, and management strategies. *Lancet Neurology*.

[28] Li Y., Yang L., Wang L. (2017). Burden of hypertension in China: a nationally representative survey of 174,621 adults. *International Journal of Cardiology*.

[29] Rothwell P. M. (2007). Atherothrombosis and ischaemic stroke. *BMJ*.

[30] Lammie G. A., Brannan F., Slattery J., Warlow C. (1997). Nonhypertensive cerebral small-vessel disease. An autopsy study. *Stroke*.

[31] Shen Z., She Q. (2018). Association between the deletion allele of Ins/Del polymorphism (rs145204276) in the promoter region of GAS5 with the risk of atherosclerosis. *Cellular Physiology and Biochemistry*.

[32] Xu L., Xia C., Xue B., Sheng F., Xiong J., Wang S. (2018). A promoter variant of lncRNA GAS5 is functionally associated with the development of osteosarcoma. *J Bone Oncol*.

[33] Tang Y., Wang Y., Wang X., Liu Y., Zheng K. (2019). A genetic variant of rs145204276 in the promoter region of long noncoding RNA GAS5 is associated with a reduced risk of breast cancer. *Clinical Breast Cancer*.

[34] Yuan J., Zhang N., Zheng Y., Chen Y. D., Liu J., Yang M. (2018). LncRNA GAS5 Indel genetic polymorphism contributes to glioma risk through interfering binding of transcriptional factor TFAP2A. *DNA and Cell Biology*.

[35] Wang Y., Wu S., Yang X., Li X., Chen R. (2019). Association between polymorphism in the promoter region of lncRNA GAS5 and the risk of colorectal cancer. *Biosci Rep*.

[36] Yan H., Zhang D. Y., Li X. (2017). Long non-coding RNA GAS5 polymorphism predicts a poor prognosis of acute myeloid leukemia in Chinese patients via affecting hematopoietic reconstitution. *Leukemia & Lymphoma*.

