# Supplementary material for: Genetic Variants of lncRNA GAS5 Contribute to Susceptibility of Ischemic Stroke among Southern Chinese Population
Source: Biomed Res Int. 2021 Apr 10;2021:6634253. doi: 10.1155/2021/6634253 (PMC8055407; doi:10.1155/2021/6634253)
Supplement: Supplementary Materials — Figure S1: the correlation between rs145204276 genotype and expression of GAS5. (A) Quantitative real-time PCR was used to examine the expression levels of GAS5 in 98 IS patients. Compared to rs145204276 ins/ins (II) carriers, rs145204276 del/del (DD) carriers had increased levels of GAS5 (∗∗P < 0.01). Expression quantitative trait loci analysis of rs145204276 with gene expression in different tissues (B), frontal cortex (C), and artery (D). eQTL: expression quantitative trait loci; NES: normalized effect size; CI: confidence interval. Table S1: logistic regression analysis for identifying risk factors of IS. [file 6634253.f1.zip › Supplemental table (1).docx]

Table S1 Logistic regression analysis for identifying risk factors of IS

| **Variables** | **B** | **OR (95% CI)** | | ***P* value** |
| --- | --- | --- | --- | --- |
| Smoking status | 0.82 | 2.27(1.53-3.38) | | < 0.001 |
| Hypertension | 1.80 | 6.05(4.49-8.13) | | < 0.001 |
| Diabetes | 0.60 | 1.82(1.17-2.82) | | 0.007 |
| Triglycerides | 0.15 | 1.16(1.06-1.27) | | 0.001 |
| HDL-cholesterol | -0.79 | 0.45(0.30-0.68) | | < 0.001 |
| LDL-cholesterol Triglycerides | 0.0060 | 1.01(0.85-1.20) | | 0.95 |
| HCY | 0.013 | 1.10(0.97-1.06) | | 0.54 |
| rs145204276 | 0.82 | 2.27(1.30-3.95) | | < 0.001 |
|  |  |  |  |  |

CI: confidence interval; HCY: Homocysteine; HDL: high-density lipoprotein;

IS: ischemic stroke; LDL:low-density lipoprotein; OR, odds ratio
