# Supplementary figures and images for: Genetic Variants of lncRNA GAS5 Contribute to Susceptibility of Ischemic Stroke among Southern Chinese Population
Source: Biomed Res Int. 2021 Apr 10;2021:6634253. doi: 10.1155/2021/6634253 (PMC8055407; doi:10.1155/2021/6634253)

## Slide 1
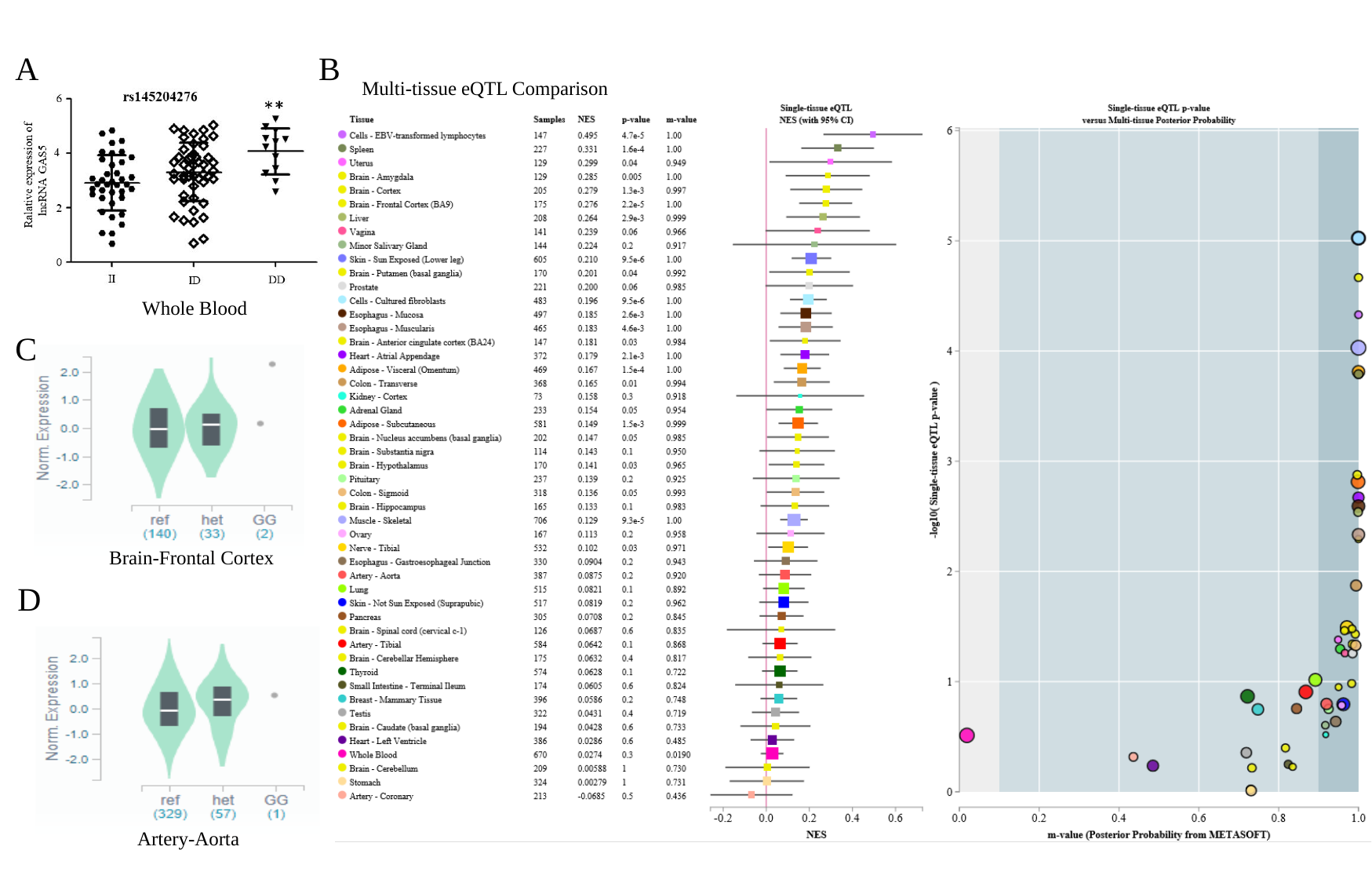

A
B
Multi-tissue eQTL Comparison
Whole Blood
C
Brain-Frontal Cortex
D
Artery-Aorta

Supplement: Supplementary Materials — Figure S1: the correlation between rs145204276 genotype and expression of GAS5. (A) Quantitative real-time PCR was used to examine the expression levels of GAS5 in 98 IS patients. Compared to rs145204276 ins/ins (II) carriers, rs145204276 del/del (DD) carriers had increased levels of GAS5 (∗∗P < 0.01). Expression quantitative trait loci analysis of rs145204276 with gene expression in different tissues (B), frontal cortex (C), and artery (D). eQTL: expression quantitative trait loci; NES: normalized effect size; CI: confidence interval. Table S1: logistic regression analysis for identifying risk factors of IS. [file 6634253.f1.zip › supplemental Figure 1.pptx]

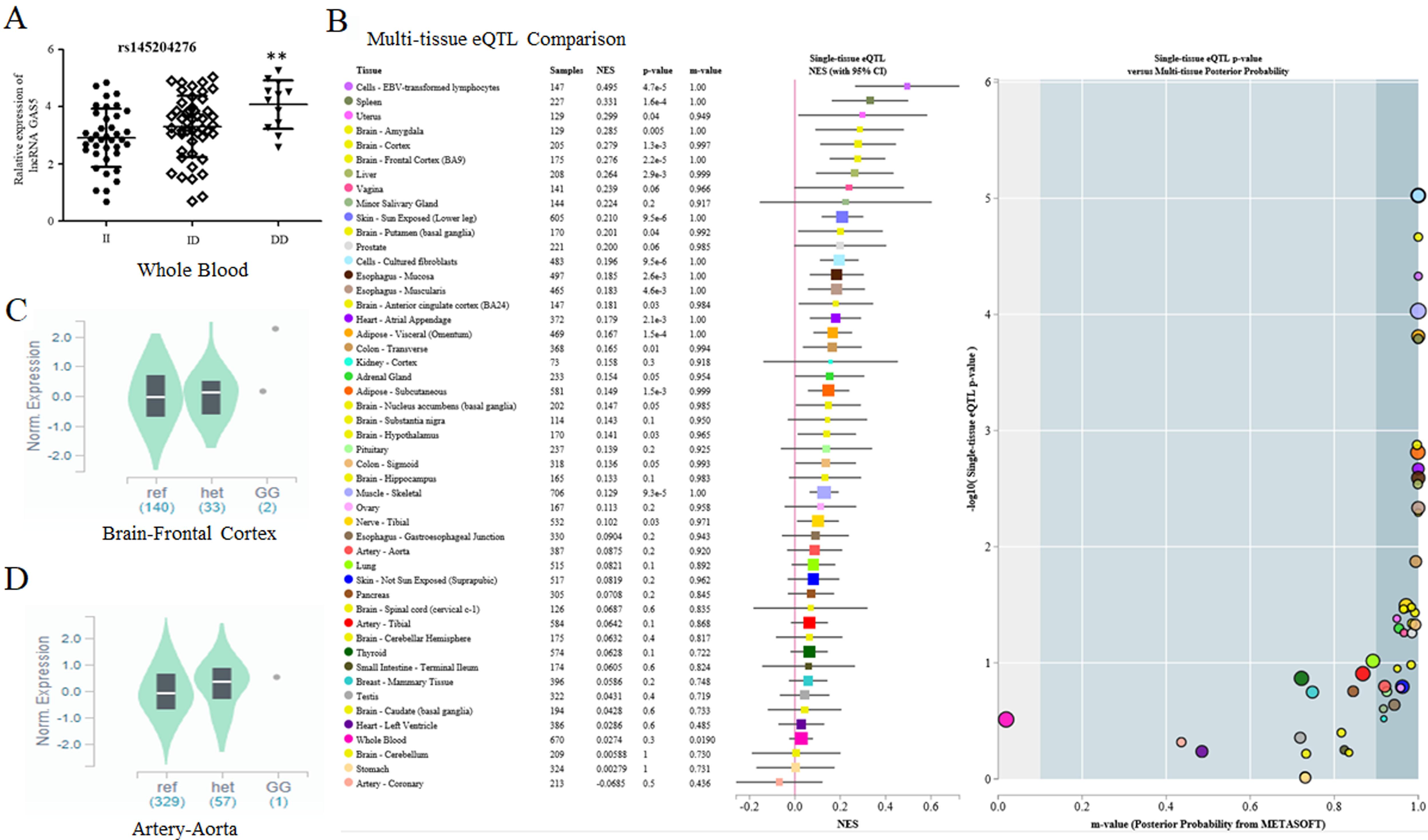

Supplement: Supplementary Materials — Figure S1: the correlation between rs145204276 genotype and expression of GAS5. (A) Quantitative real-time PCR was used to examine the expression levels of GAS5 in 98 IS patients. Compared to rs145204276 ins/ins (II) carriers, rs145204276 del/del (DD) carriers had increased levels of GAS5 (∗∗P < 0.01). Expression quantitative trait loci analysis of rs145204276 with gene expression in different tissues (B), frontal cortex (C), and artery (D). eQTL: expression quantitative trait loci; NES: normalized effect size; CI: confidence interval. Table S1: logistic regression analysis for identifying risk factors of IS. [file 6634253.f1.zip › supplemental Figure 1.tif]
